# Developmental plasticity to pond drying has carryover costs on metamorph performance

**DOI:** 10.1093/conphys/coaf008

**Published:** 2025-02-13

**Authors:** Nicholas C Wu, Nien-Tse Fuh, Amaël Borzée, Chi-Shiun Wu, Yeong-Choy Kam, Ming-Feng Chuang

**Affiliations:** Hawkesbury Institute for the Environment, Western Sydney University, Science Rd, Richmond 2753, NSW, Australia; Department of Life Science, Tunghai University, No. 1727, Sec. 4, Taiwan Blvd, Xitun Dist, Taichung 407224, Taiwan; Laboratory of Animal Behaviour and Conservation, College of Biology and the Environment, Nanjing Forestry University, No.159 Longpan Rd, Xuanwu Dist, Nanjing 210037, China; Department of Life Science, Chinese Culture University, No. 55, Hwa-Kang Rd, Yang-Ming-Shan, Taipei 11114, Taiwan; Department of Life Science, Tunghai University, No. 1727, Sec. 4, Taiwan Blvd, Xitun Dist, Taichung 407224, Taiwan; Department of Life Sciences, National Chung Hsing University, No. 145, Xingda Rd, South Dist, Taichung 402202, Taiwan; Global Change Biology Research Center, National Chung Hsing University, No. 145, Xingda Rd, South Dist, Taichung 402202, Taiwan

**Keywords:** Amphibian, climate change, drought, fitness, life history, locomotion, tadpole

## Abstract

Increasing variable hydroperiods may leave ectotherms with complex life cycles more vulnerable to the impacts of environmental drying. While developmental plasticity may enable some species to escape drying ponds, this plasticity might result in trade-offs with performance and subsequent fitness in adults. Here, we used rice paddy frogs (*Fejervarya limnocharis*) to test how pond drying influences the developmental plasticity of tadpoles, and the resulting carryover effects on body size and jumping performance. We predicted that tadpoles under simulated drought conditions (2–0.25 cm depth) compared to low stable water level conditions (0.25 cm depth) would develop faster, and the resulting metamorphs would be smaller and exhibit lower jumping performance. We show that tadpoles in drying conditions had a faster developmental rate than tadpoles in stable low water level treatments. The size of metamorphs from the drying treatment was similar to the high-water treatments (2 cm depth), but maximum jumping distance of individuals from the drying condition was lower than that of the high-water treatment. These results indicate that drying conditions for *F. limnocharis* increase development rate without a reduction in size at metamorphosis, but with poorer mass-independent locomotor performance, which can potentially impact their survival.

## Introduction

The ability for animals with water-dependent life stages to adjust their developmental rate under variable periods of water availability enables them to survive in highly dynamic environments (Richter-Boix *et al.,* 2011). This developmental plasticity is hormonally and neurologically modulated, influencing growth rate and time to metamorphosis in response to environmental cues (Denver, 2009, 2013). Amphibians vary greatly in developmental rates, which can change in response to fluctuations in temperature, presence of predators, salinity and desiccation (Newman, 1988; Lai *et al.,* 2002; Benard, 2004; Wu and Kam, 2009; Higginson and Ruxton, 2010; Thompson and Popescu, 2021; Sinai *et al.,* 2022). Environmental conditions that cause amphibians to shorten developmental periods often result in smaller body size at metamorphosis (Forster *et al.,* 2011; Audzijonyte *et al.,* 2019). This reduction in size arises from changes in neuroendocrinological pathways, such as thyroid-mediated and corticosteroid responses, as environmental stressors directly impact growth (Kirschman *et al.,* 2017; Ruthsatz *et al.,* 2020). Smaller body size can negatively affect fitness, reducing locomotor performance, predator avoidance, conspecific interaction, reproduction, to increasing susceptibility to disease (Gervasi and Foufopoulos, 2008; Cabrera-Guzmán *et al.,* 2013; Malerba *et al.,* 2018; Wu *et al.,* 2018; Brannelly *et al.,* 2019). Consequently, external stressors that prompt faster development to escape stressful conditions, along with the associated smaller body size at metamorphosis, may have long-term consequences for fitness and survival (Berven, 1990; DeWitt *et al.,* 1998; Tarvin *et al.,* 2015; Thompson and Popescu, 2021).

Studies on the carryover effects of pond drying are increasingly important as thermal extremes continue to rise (Gervasi and Foufopoulos, 2008; Ohmer *et al.,* 2023). While drying has been shown to shorten the developmental period, resulting in smaller metamorphs (Tejedo *et al.,* 2010; Richter-Boix *et al.,* 2011; Brannelly *et al.,* 2019), the potential fitness trade-offs associated with smaller metamorph size may have serious implications for survival and reproductive success. Recent studies have demonstrated that drought conditions and smaller body size contribute to increased disease susceptibility (Wu *et al.,* 2018; Kohli *et al.,* 2019; Le Sage *et al.,* 2022; Buttimer *et al.,* 2024). Given the inherent vulnerability of amphibians to environmental drying due to their sensitive skin and water-dependent life stage (Feder and Burggren, 1992; Liedtke *et al.,* 2022), understanding how different species respond to pond drying is crucial. Such insights can inform targeted management efforts for species most at risk from increasing climate change and human-mediated impacts, such as habitat modification (Cheng *et al.,* 2023; Smith *et al.,* 2023).

**Figure 1:**
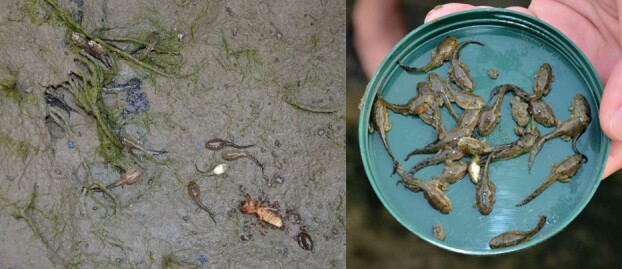
Field observations of *F. limnocharis* tadpoles in drying ponds (<1 cm water) during the month of June 2014. Tadpoles were of late development (Gosner Stage 38–44). Authors noted most were still alive, while some dead were observed in the outer perimeter of the ditch. Photo by M.F.C.

We examined the carryover costs in the rice paddy frog (*Fejervarya limnocharis*), a species that breeds in various water bodies, including temporary ones (Fig. 1), when developing under pond-drying conditions. *Fejervarya limnocharis* exhibits varying developmental plasticity in response to factors such as temperature and salinity (Kuan and Lin, 2011; Wu *et al.,* 2012), making it an ideal model for understanding developmental plasticity and carryover responses to low water levels and pond drying. Elucidating the performance cost experienced by metamorphs developing in drying ponds is crucial for predicting how animals with complex life cycles cope with increasing climate extremes. Drying events, such as drought, can affect amphibians at individual, population and community levels. We specifically tested how rearing tadpoles under decreasing water levels (pond drying) compared to stable water levels influences tadpole growth rate, body size at metamorphosis and jumping performance after metamorphosis.

## Materials and Methods

### Animal collection and maintenance

Egg clutches of similar developmental stages were collected in June 2014 from a ditch (maximum depth of 5 cm) near a rice field in Miaoli County, Taiwan (24.628123°N, 120.761152°E). Tadpoles were also observed in the ditch, where most were alive, while some were freshly dead around the outer perimeter (Fig. 1). The eggs were transported to the laboratory facilities at Tunghai University and kept in containers filled with aged, filtered water (sourced from groundwater) with a pH from 7.2 to 7.5. The water was treated using a three-stage filtration system (iSpring US31, iSpring Water Systems, GA, USA). After hatching, tadpoles were not fed as they relied on reserves from their yolk sacs until their external gills disappeared (Gosner Stage 25; Gosner, 1960). Once the tadpoles reached Gosner Stage 25, they were randomly assigned to one of five treatments (*n* = 11–12 per treatment). Each tadpole was reared individually in a 10.5 × 7.5 × 4.5 cm plastic container with varying water depths at ~25°C on a 12 D:12 L photoperiod until metamorphosis. This set-up excluded the influence of crowding on development under differing water levels. Tadpoles were fed *ad libitum* with boiled amaranth or spinach daily until satiated (with leftovers present). Water in the containers was renewed twice a week until all tadpoles completed metamorphosis. The metamorphs (hereafter referred to as froglets) were maintained in plastic containers and released at their original habitat within 4 days. All experiments were conducted in accordance with the Institutional Animal Care and Use Committees of Tunghai University (IACUC approval No. 103–16). All animals were returned to their collection site following the experiments.

### Experimental design

Five treatments were used to distinguish the effects of water depth and drying conditions. For four treatments, containers were filled with a fixed depth of either 0.25, 0.5, 1 or 2 cm of aged, filtered water. In the fifth treatment (representing pond drying), the containers were initially filled to a depth of 2 cm on Day 1, then reduced to 1 cm on Day 8, 0.5 cm on Day 15 and finally filled to 0.25 cm on Day 21, where they were maintained until all tadpoles completed metamorphosis. Water level treatments were based on field measurements in rice paddies. The larval period, or time to metamorphosis (in days), was recorded for all treatments.

### Body size and jumping performance measurements

Photos of each tadpole was taken weekly with a scale, from the beginning of the experiment until the tadpoles reached developmental stage 42 (Gosner, 1960), when tail absorption begins. The photos were analysed using Image J (Rueden *et al.,* 2017) to measure the total length (in centimetres) of the tadpoles (snout-to-tail). Once the tadpoles had metamorphosed, snout-vent length (SVL; in centimetres) was measured as the size of the metamorph. This was done by placing each froglet on a petri dish with a lid, aligning the dish with a scale, and photographing the underside of the froglet when it was flat on the dish, ensuring the SVL was parallel to the scale. Growth rate (in centimetres per day) was calculated as the difference between the final and initial length divided by the number of days between the final and initial measurements. Body mass was not measured to avoid unnecessary stress on the tadpoles, as drying the skin to remove excess water could be harmful. However, SVL and body mass are known to be highly correlated (Takahara *et al.,* 2008; Santini *et al.,* 2018).

The maximum jumping distance (in centimetres) of each froglet was measured following the method of Hsu *et al.* (2012). Excess moisture on the froglet was first gently dampened before they were placed on a clean, flat surface. To elicit jumping, the vent area of each froglet was lightly touched with a probe to induce an escape response. Each froglet performed 10 jumps, and the maximum distance for each individual was used for analysis. Summary variables are provided in Supplementary Table S1.

### Statistical analysis

All analyses were performed in *R* (R Core Team, 2021). Data are presented as means ± standard deviation (SD) or individual data points. The significance threshold (α) was set at 0.05 for all statistical tests.

#### Tadpole growth

The independent variable total length (in centimetres) between treatment groups and across days of exposure (dependent variable) was analysed via a linear mixed-effects model using the ‘lmer’ function from the *lme4* package (Bates *et al.,* 2014), with water level groups and days of exposure as interactive effects, and individual identity to account for the random intercept and slope of the repeated measurements within individuals. To test the differences between slopes (across exposure days), the ‘lstrends’ function from the ‘*emmeans* package (Lenth, 2024) was used to extract the slopes, and the ‘pairs’ function compared the slopes across treatments.

#### Life history traits

The independent variables growth rate (in centimetres per day), larval period (days), metamorph size (in centimetres) and maximum jumping distance (in centimetres) across each water level treatment (dependent variable) were analysed with linear effects models using the base *R* ‘lm’ function. To test for interaction between treatments, a chi-squared test with Holm adjustment method was performed using the ‘contrast’ function from the *emmeans* package.

## Results

### Developmental rate and period

**Figure 2:**
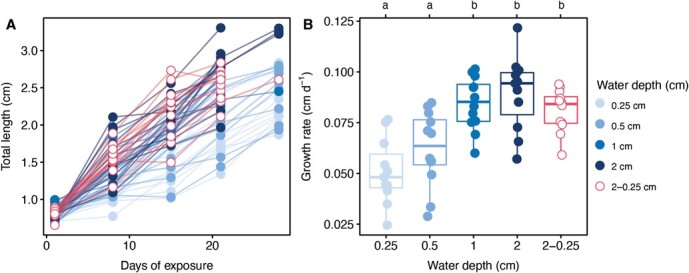
(**A**) Relationship between *F. limnocharis* tadpole total length (in centimetres) and water depth (in centimetres) across exposure days. Data points and lines represent tracked individual growth. (**B**) The growth rate (in centimetres per day) across different water depth treatments. Data were presented as individual data points and summarized as boxplots. Boxplots show 25th and 75th percentile, the centreline is the 50th percentile and outer lines represent the max/min values (within the 1.5 × IQR). Significant differences between treatments were represented as lowercase letters. Full statistical summary provided in electronic Supplementary Tables S2–S3.

**Figure 3:**
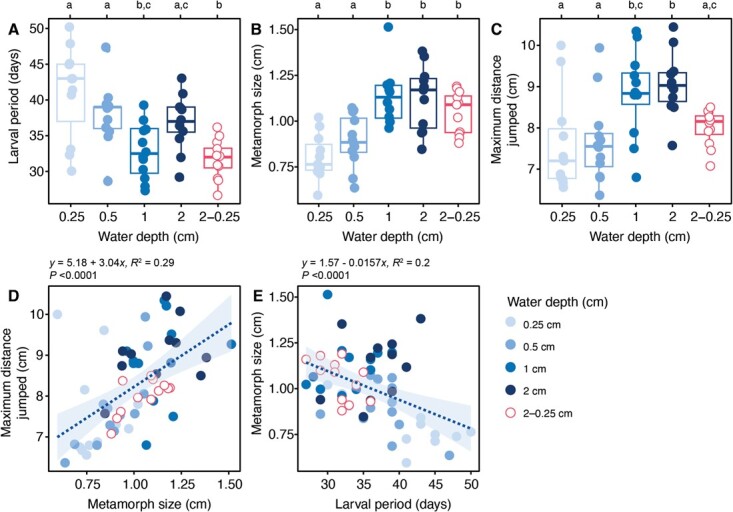
(**A**) Larvaljjjjjjjjjjperiod (days), (**B**) metamorph size (SVL, in centimetres) and (**C**) maximum jumping distance (in centimetres) of *F. limnocharis* under different water depth treatments. (**D**) Relationship between metamorph size (in centimetres) and maximum distance jumped (in centimetres), and (**E**) larval period (days) and metamorph size (in centimetres). Data in A–C were presented as individual data points and summarized as boxplots. Boxplots show 25th and 75th percentile, the centreline is the 50th percentile and outer lines represent the max/min values (within the 1.5 × IQR). Dash lines in D–E represent linear regression (including regression equation, *R*^2^ and significance). Significant differences between treatments were represented as lowercase letters. Full statistical summary provided in electronic Supplementary Tables S4–S6.

The tadpole total length over time differed between water depth treatment (Fig. 2a). This translated into differences in the developmental rate (Fig. 2b). Tadpoles from the lower water level treatments (0.25 and 0.5 cm) had significantly lower developmental rates compared to those at relatively higher water levels (1 and 2 cm; Supplementary Tables S2 and S3). On average, the tadpole growth rate was 1.7 times lower in the 0.25-cm treatment compared to the 2-cm treatment (0.05 ± 0.02 cm d^−1^ and 0.09 ± 0.02 cm d^−1^, respectively, Supplementary Table S1). The developmental rate under the simulated pond-drying treatment (2–0.25 cm) did not significantly differ from the higher water level treatments (*t*_53_ = 1.32, *P* = 0.668; Fig. 2a and b). The larval period, or time in treatments before metamorphosis (days), was longest in the low water level treatment (0.25 cm; 43 ± 6.6 days) and shortest in the pond-drying treatment (2–0.25 cm; 32.9 ± 2.6 days; *t*_53_ = 5.06, *P* ≤ 0.0001; Fig. 3a).

### Metamorph size and jumping performance

The SVL measurements of froglets were the smallest in the 0.25-cm treatment (0.8 ± 0.12 cm), and the largest in the 2-cm treatment (1.13 ± 0.18 cm; *t*_53_ = 5.33, *P* < 0.0001; Fig. 3b). The maximum jumping distance followed a similar trend, with the shortest distance recorded in the 0.25-cm treatment (7.6 ± 1.19 cm), and the longest in the 2-cm treatment (9.06 ± 0.78 cm; *t*_53_ = −3.66, *P* = 0.001; Fig. 3c). The larval period (Supplementary Table S4) and metamorph SVL (Supplementary Table S5) in the decreasing water level treatment did not significantly differ from those in the high water level treatments. However, the maximal jumping distance was significantly lower in the decreasing water level treatment compared to the high water level treatment (Fig. 3c, Supplementary Table S6). The similarity in trends between froglet size and maximum jumping distance can be partially explained by the relationship between metamorph size and maximum distance jumped. As froglet size increased, the maximal jumping performance also increased (*R*^2^ = 0.29; *P* < 0.0001; Fig. 3d). In contrast, the relationship between larval period and the subsequent metamorph size was negatively correlated. As the larval period increased, metamorph size decreased (*R*^2^ = 0.2; *P* < 0.0001; Fig. 3e).

## Discussion

Environmental drying events, such as drought, are increasingly prominent climatic stressors with the potential to impact amphibian communities, prompting management efforts to preserve habitats with water bodies essential for amphibian development. Our results showed that tadpoles developing under pond-drying conditions experienced shorter larval period and reached similar body size after metamorphosis compared to those in the high water level treatment. However, they exhibited a shorter maximum jumping distance, suggesting carryover costs on performance resulting from a faster developmental rate in response to drought.

The accelerated development rate relative to the low water level treatment in our study aligned with the existing literature showing that pond-drying conditions elicit shorter larval periods (Richter-Boix *et al.,* 2011; Gomez-Mestre *et al.,* 2013; Brannelly *et al.,* 2019). Interestingly, constant low water level conditions reduced growth rate and extended the time to metamorphosis. The difference in growth rate trajectories between drying-induced and stable low water levels in our study may reflect the influence of external cues (e.g. decreasing water levels) and the timing of the stress response modulating growth rate. Exposure to environmental stressors earlier in the development has been shown to alter growth and development in response to variables such as high density (Denver, 1997; Glennemeier and Denver, 2002), higher temperatures and the presence of predators (Albecker *et al.,* 2023). Tadpoles that show accelerated development are typically subjected to stressors later in development (Gervasi and Foufopoulos, 2008; Denver, 2013). Differences in developmental rate may arise from the balance between allocating energy for somatic growth and initiating the neuroendocrinological response to stress (de Souza and Kuribara, 2006; Ruthsatz *et al.,* 2019; Pfab *et al.,* 2020). During early development, tadpoles may allocate most of their energy intake for somatic growth, especially for ephemeral species employing consumption-driven growth tactics (Pujol-Buxó *et al.,* 2016). When subjected to an external stressor during early development, such as under low-water conditions, energy resources may shift towards the production of stress hormones, which in turn have growth-inhibiting effects. Conversely, under conditions of decreasing water levels, the timing of the stress response may facilitate appropriate modulation of thyroid signalling, accelerating development towards metamorphosis (Denver, 2013). As the unpredictability of drought extremes increase globally (Trenberth *et al.,* 2014), amphibians may face greater challenges in responding adaptively and efficiently modulating their growth rate, such as when pond drying occur sooner during early development.

Small size at metamorphosis is associated with reduced fecundity (Márquez-García *et al.,* 2010), a depressed immune system and increased susceptibility to diseases (Gervasi and Foufopoulos, 2008; Wu *et al.,* 2018), delayed reproductive age (Semlitsch *et al.,* 1988) and decreased locomotor ability (Nicieza *et al.,* 2006). Locomotion is of particular interest because performance influences escape potential from predators, dispersal and foraging efficiency (Biewener and Patek, 2018; Wu and Seebacher, 2022), which, in turn, affects an animal’s overall fitness. The relationship between the rate of development, size after metamorphosis and post-metamorphic performance is well demonstrated (Emerson *et al.,* 1988; Alvarez and Nicieza, 2002; Nicieza *et al.,* 2006; Capellán and Nicieza, 2007; Thompson and Popescu, 2021). We found that tadpoles exposed to pond-drying conditions (2–0.25 cm) and low water levels (0.25 cm), exhibited decreased maximal jumping distance compared to those in the 2-cm water depth treatment. However, size at metamorphosis differed between the drying condition and low water level treatment. Under constant low-water stress, energy allocation for growth, somatic maintenance, and stress response within a limited resource budget has been proposed to be the primary driver of variability in development time and subsequent body size (Richter-Boix *et al.,* 2011; Gomez-Mestre *et al.,* 2013).

The reduced growth rate observed during development under low-water conditions relative to the 2-cm treatment may reflect a shift in energy allocation away from growth and towards the stress response. This shift likely reduces available energy for cellular rearrangement during metamorphosis, an energetically demanding transformation (Pandian and Marian, 1985), resulting in smaller froglet size, as observed in this study. Interestingly, body size under drying conditions did not decrease significantly compared to the high-water treatment (2 cm). However, the cost of accelerating development time on locomotor performance may be linked to reductions in limb morphology. For example, proportionally shorter limbs have been observed under drying exposure (Gomez-Mestre *et al.,* 2010; Gomez-Mestre *et al.,* 2013), potentially explaining the lower maximal jumping performance observed in our study. Thus, the developmental cost in pond-drying conditions may have long-term carryover effects on the animal’s fitness and survival. Future studies should focus on quantifying field activity in populations experiencing different developmental trajectories in response to pond drying to determine overall activity costs associated with developmental plasticity.

Under the impact of global warming, environmental evapotranspiration rates are expected to intensify (Dai, 2013), increasing the desiccation risk for animals that rely on water bodies. Although we identified the capability of developmental plasticity in response to pond drying in *F. limnocharis*, we observed tadpoles drying out before metamorphosis in the field (Fig. 1). Even though adaptive plasticity enhances survival under unstable and dynamic environments, the rapidly changing climate and frequent extreme climatic events are undoubtedly exceeding the response capacity of some animals (Radchuk *et al.,* 2019). We encourage future studies to examine the breadth of developmental plasticity in amphibians, correlating it with environmental niches and modelling how specific habitats susceptible to drought that may impact particular amphibian communities (Richter-Boix *et al.,* 2011). For example, habitat degeneration and climate change are major threats to amphibian populations (Luedtke *et al.,* 2023). Targeted management efforts of high-risk areas, such as agricultural and urban landscapes, through increasing the hydroperiod of small water bodies, will help alleviate the developmental stress caused by accelerated pond drying. South America and Southeast Asia host the highest amphibian diversity (Fritz and Rahbek, 2012), but these regions are at risk of increasing drought events, particularly in the Amazon and Atlantic Forest where droughts have historically been extremely rare (Phillips *et al.,* 2009; Wu *et al.,* 2024). Not all amphibians exhibit high developmental plasticity (Richter-Boix *et al.,* 2011), thus, species from historically more stable climatic environments are at greater risk from drought events, especially populations in more urbanized regions where deforestation and land modification exacerbate vulnerability (Merckx *et al.,* 2018).

## Supplementary Material

Web_Material_coaf008

## Data Availability

Data and *R* codes are publicly available in the GitHub repository: https://github.com/nicholaswunz/tadpole-pond-drying
